# Secondary infection profile after snakebite treated at a tertiary referral center in the Brazilian Amazon

**DOI:** 10.1590/0037-8682-0244-2021

**Published:** 2022-02-25

**Authors:** Viviane Kici da Graça Mendes, Handerson da Silva Pereira, Ignês Cruz Elias, Gean Souza Soares, Monica Santos, Carolina Talhari, Marcelo Cordeiro-Santos, Wuelton Marcelo Monteiro, Jacqueline de Almeida Gonçalves Sachett

**Affiliations:** 1 Universidade do Estado do Amazonas, Programa de Pós-Graduação em Ciências Aplicadas à Dermatologia, Manaus, AM, Brasil.; 2 Fundação Alfredo da Matta, Diretoria de Ensino e Pesquisa, Manaus, AM, Brasil.; 3 Universidade do Estado do Amazonas, Programa de Pós-Graduação em Medicina Tropical, Manaus, AM, Brasil.; 4 Fundação de Medicina Tropical Dr. Heitor Vieira Dourado, Manaus, AM, Brasil.

**Keywords:** Snakebite, Secondary infection, Bothrops

## Abstract

**Background:**

*Bothrops* envenomations can often lead to complications, such as secondary infections.

**Methods:**

This cross-sectional study analyzed the medical records of all patients diagnosed with snakebite.

**Results:**

A total of 127 patients were included. Clindamycin was the most commonly prescribed antibiotic, with 105 patients (82.7%) receiving it as the primary antibiotic regimen. In 31 (24.4%) individuals, the first-choice antibiotic did not cease the infection.

**Conclusions:**

Secondary infection is an important complication resulting from snakebites, and evidence-based management of this complication can contribute to better clinical outcomes.

In Brazil, snakes of the genus *Bothrops* cause the greatest number of envenomations, with complications such as bleeding, acute renal failure, and secondary bacterial infections^1^ ^,^ ^2^. Envenomations caused by the genus *Lachesis* are very similar to those caused by *Bothrops*; thus, complications are also similar^3^ ^,^ ^4^. In the Brazilian Amazon, abscesses and cellulitis appear at the site of the bite in approximately 40% of patients bitten by *Bothrops* ^2^. Secondary infections from snakebites may result from invasion of the tissue by the patients’ skin microbiota and/or oral microbiota of the perpetrating snake^5^ ^,^ ^6^. 

There are no established criteria for universal diagnosis of secondary bacterial infection^7^. Studies have shown the presence of clinical signs of severity, such as phlogistic signs, namely heat, flushing, pain, and vesicles, necrosis, and purulent discharge. Patients with moderate or severe snakebite, with larger tissue injury, are considered more susceptible to the development of secondary bacterial infection^2^ ^,^ ^7^. Leukocytosis and elevation of transaminases and C-reactive protein levels are laboratory findings that help in the diagnosis of secondary infections from snakebites^2^. 

Antibiotics with a large antibacterial spectrum, such as third-generation cephalosporins, piperacillin-tazobactam, and ciprofloxacin, are active antibiotics for treating secondary infections caused by snakebites^7^ ^,^ ^8^. However, as yet, there is no recommendation to use of an antibiotic for the prevention of these infections after a snakebite. Although amoxicillin clavulanate can be used for animal bites^8^, it has not been shown to be effective for snake bites^2^. Thus, this study aimed to characterize local secondary infections from snakebites and treat them at a referral center in the Brazilian Amazon.

A cross-sectional study was conducted at the Fundação de Medicina Tropical Dr. Heitor Vieira Dourado (FMT-HVD), Manaus, Amazonas, Brazil, which is a tertiary referral unit that is responsible for the treatment of an average of 200 snakebites annually in the Amazonas state, of which 40% present secondary infections^2^. The sample was composed of patients who were victims of snakebite, treated from January 2017 to December 2018, and developed secondary bacterial infections. Epidemiological, clinical, and laboratory data were also collected. A database was constructed, and a descriptive analysis of the absolute and relative frequencies was performed. This study was approved by the Research Ethics Committee of the Universidade do Estado do Amazonas (approval number: 713.140). 

Secondary infection after a *Bothrops* snakebite was defined as the presence of cellulitis and/or abscesses^8^ up to 48 h after admission. Cellulitis was defined as the presence of local signs of inflammation (erythema, edema, heat, and pain) associated with fever, leukocytosis, lymphangitis, and/or lymphadenitis, and the abscess was characterized by the presence of an individualized, floating lesion with purulent or seropurulent discharge^2^.

We analyzed 545 cases of snakebite treated between 2017 and 2018 at the FMT-HVD. Of these, 127 (23.3%) developed a secondary bacterial infection. Most cases involved men (101; 79.5%); 64 (50.4%) patients were aged between 16 and 45 years, and 28 (22.0%) were agricultural workers (Table 1). The lower limbs were the most affected body region (111 patients; 87.4%), and most bites occurred in rural areas (104; 81.9%). In 110 (86.6%) patients, there was information regarding the time elapsed between the bite and hospital admission; of these, 67 (60.9%) were seen between 3 and 6 h after the snakebite. Regarding the severity of envenomation, 62 (48.8%) patients were diagnosed with moderate envenomation and 110 (90.2%) received antivenom treatment. Edema and pain were the most frequently observed local clinical signs, reported by 121 (95.3%) and 117 (92.1%) patients, respectively. In the medical records of 79 (61.5%) patients, there was information on skin lesions, and of these, 27 (21.3%) patients had blisters. Purulent and hemorrhagic exudates were observed in 17 (13.4%) and 17 (13.4%) patients, respectively. Necrosis was observed in 41 (32.3%) patients (Table 1).

**TABLE 1:** Distribution of cases of snakebite with secondary infection according to sociodemographic variables and specific information regarding the bite.

Variable	Total n (%)
**Sex**	
Male	101 (79.5)
Female	26 (20.5)
**Age (years)**	
0-15	20 (15.7)
16-45	64 (50.4)
46-60	25 (19.7)
>60	18 (14.2)
**Occupation**	
Farmer	28 (22.0)
Student	19 (15.0)
Fisher	4 (3.1)
Driver	2 (1.5)
Others	8 (6.4)
Not informed	66 (52.0)
**Location of the snakebite**	
Upper limbs	13 (10.2)
Lower limbs	111 (87.4)
Head	2 (1.6)
Not informed	1 (0.8)
**Zone of occurrence**	
Rural	104 (81.9)
Urban	5 (3.9)
Periurban	8 (6.3)
Not informed	10 (7.9)
**Time until treatment (h)**	
0-6	67 (60.9)
7-24	31 (28.2)
>24	12 (10.9)
**Severity classification**	
Mild	14 (11.0)
Moderate	62 (48.8)
Severe	28 (22.0)
Not informed	23 (18.2)
**Antivenom**	
Yes	109 (85.8)
No	18 (14.2)
Number of vials*	
2-4	15 (13.7)
5-8	62 (56.9)
>9	32 (29.4)
Local manifestations**	
Edema	121 (95.3)
Pain	117 (92.1)
Erythema	79 (62.2)
Systemic manifestations**	
Vomiting	17 (13.4)
Blurred vision	5 (3.9)
Fever	5 (3.9)
Basic injury**	
Erythematous plaque	6 (4.7)
Exulceration	17 (13.4)
Blister	27 (21.3)
Nodule	2 (1.6)
Pustule	3 (2.4)
Ulcer	23 (18.1)
Exudate from the lesion**	
Purulent	17 (13.4)
Hemorrhagic	17 (13.4)
Serous	7 (5.5)
Seropurulent	8 (6.3)
Serous/hemorrhagic	16 (12.6)
Not informed	2 (1.6)
**Type of tissue in the lesion**	
Necrosis	41 (32.3)
Granulation	26 (20.5)
Not informed	60 (47.2)
**Topical product use**	
Yes	9 (7.1)
Not given	118 (92.9)

*The number of vials was calculated only for patients who received antivenom therapy. **For these manifestations, patients may present one or more variables simultaneously.

In five (3.9%) patients, culture examination was performed, and only one presented with bacterial growth (*Proteus mirabilis)*. Clindamycin was the first-choice drug in 105 (82.7%) patients and only 18 (17.1%) required another second-choice antibiotic. Treatment with clindamycin resulted in fewer therapeutic failures than other antimicrobial regimens (odds ratio, 9.059; confidence interval, 3.239-24.92; p<0.001). In 31 (24.4%) individuals, the first-choice antimicrobial treatment was not effective, and a second therapeutic regimen was used. In this second treatment regimen, clindamycin was also the most commonly used drug (nine patients; 29.0%) (Table 2). Most patients required abscess drainage (76.5%). Among the laboratory tests performed on admission, leukocyte and alanine aminotransferase and aspartate aminotransferase levels were elevated (Table 3).

**TABLE 2:** Antibiotics used to treat secondary infection.

Antibiotic	Primary regimen	Secondary regimen
	n=127 (%)	N=31 (%)
Clindamycin (600 mg EV every 6 h for 7 days, OR 600 mg every 8 h for 10 days)	105 (82.7)	9 (29.0)
Ceftriaxone (1 g EV every 12 h for 7 days OR 2 d EV every 12 h for 10 days)	6 (4.7)	5 (16.1)
Cefalotin (2 g EV every 6 h for up to 7 days OR 800 mg EV every 6 h for 7 days, OR 470 mg EV every 6 h for 7 days)	4 (3.2)	4 (12.9)
Amoxicillin + clavulanate (17 mL PO every 12 h for 7 days)	3 (2.3)	2 (6.5)
Ampicillin + sulbactam (1.5 g EV every 8 h for 10 days)	3 (2.3)	2 (6.5)
Cefalexin (4 ml PO every 6 h for 7 days)	2 (1.6)	2 (6.5)
Cefalotin + cefalexin (600 mg EV every 6 h for 10 days + Cefalexin 4 ml PO every 6 h for 7 days)	1 (0.8)	-
Ciprofloxacin (500 mg PO every 12 h for 5 days OR 500 mg PO every 12 h for 7 days)	1 (0.8)	2 (6.5)
Gentamicin (80 mg EV every 8 hours for 7 days, OR 95 mg IM every 24 h for 7 days)	1 (0.8)	2 (6.5)
Metronidazole (500 mg EV every 8 h for 10 days)	1 (0.8)	1 (3.2)
Penicillin G benzathine (1.200.000 IU IM every 6 h for 7 days)	-	1 (3.2)
Piperacillin + tazobactam (4 g + 0.5 g EV every 6 h for 14 days)	-	1 (3.2)

**Key:** EV: endovenous; PO: per os; IM: intramuscular.

**TABLE 3:** Laboratory tests performed on patients with secondary infection after a snakebite.

Laboratory tests	Mean (±SD)
Erythrocytes (cells ×10^6^/mm^3^)	5.9 (±10.5)
Hemoglobin (g/dL)	13.1 (±2.7)
Hematocrit (%)	39.8 (±5.8)
Leukocytes (cells ×10^3^/mm^3^)	12.0 (±7.0)
Platelets (mm^3^)	250.3 (±171.3)
Creatinine (mg/dL)	1.1 (±1.2)
Aspartate aminotransferase (IU/L)	52.1 (±52.1)
Alanine aminotransferase (IU/L)	50.3 (±71.2)
Urea (mg/dL)	34.6 (±17.8)
Clotting time (min)	4.1 (±4.1)

**Reference values:** erythrocytes: 4.7-6.1 million/mm3; hemoglobin: 13.0-16.0 g/dL; hematocrit: 40.0-52.0%; leukocytes: 4,000-10,800/mm3; rod cells: 1-5%; lymphocytes: 20.0-51.1%; monocytes: 1.70-9.30%; platelets: 130,000-400,000/mm3; creatinine: 0.5-1.2 mg/dL; aspartate aminotransferase: 2-38 IU/L; alanine aminotransferase: 2-44 IU/L; urea: 10-45 mg/dL.

In this study, secondary bacterial infection was observed in 127 (23.3%) cases analyzed, which was slightly lower than the frequency found in previous studies in the region. It is known that infection is also related to the oral microbiota of snakes, which comprises a wide variety of aerobic and anaerobic microorganisms, including Enterobacteriaceae, principally *Morganella* spp.*, Escherichia coli, Streptococcus, Aeromonas* spp*., Staphylococcus aureus,* and *Clostridium* spp.^9^. 

In a more comprehensive study^10^, isolation and bacterial characterization were performed on samples of 99 abscesses from snakebites caused by the genus *Bothrops* in Goiás, Brazil, between 1984 and 1988. Of the 15 isolated microorganisms, *Morganella morganii*, *Escherichia coli*, and *Providencia* sp. prevailed, and these were present in 44.4%, 20.2%, and 13.1% of the samples, respectively. In the Amazonas state, Brazil, culture of samples from 11 patients, which included six positive cases, presented *Morganella morganii* in five cases and *Staphylococcus aureus* in one case^2^. Another study described the oral bacterial flora of the *Bothrops jararaca* from the state of São Paulo, with the most frequent microorganisms being *Streptococcus*, *Enterobacter* sp*., Providencia rettgeri, Providencia* sp*., Escherichia coli, Morganella morganii,* and *Clostridium* sp*.* ^9^. 

The difference between the pathogenic microorganisms found in the oral microbiota of snakes may have contributed to the difference in the frequency of infections found in this study, since a correlation between the oral microbiota of venomous snakes (especially those of the genus *Bothrops*) and bacteria found in secondary infections and abscesses has been suggested^9^ ^,^ ^10^. However, there is evidence that the infection may not necessarily be associated with the oral microbiota of the snake on its own^2^. 

In the Amazon region, the distance to medical facilities is a major obstacle because of the geographical peculiarities of the region; thus, patients are more prone to develop complications^11^. Moreover, 31.6% of patients were treated ≥ 7 h after the bite, and previous studies have revealed that this factor may contribute to the incidence of secondary bacterial infection, since the longer the time elapsed between the snakebite and treatment, the greater the risks of these complications^11^ ^,^ ^12^. This results in impaired access to health services for the provision of antivenom treatment and often leads to the use of traditional medicine as a treatment option^13^. The use of traditional medicine often includes the use of a tourniquet and traditional practices, such as the use of roots, fruits, leaves, tree bark, seeds, and other foods and local manipulation in the form of incisions and suction, which may also contribute to the development of secondary infections^2^ ^,^ ^11^ ^,^ ^13^.

In this study, only a few patients underwent culture examination, and only one patient presented with bacterial growth (*Proteus mirabilis*). *P. mirabilis* is an aerobic gram-negative bacterium that is found in material collected from wounds caused by a snakebite and in the oral cavity of snakes^6^ ^,^ ^7^ ^,^ ^14^. The fact that cases of secondary infection have cellulitis as the most frequent clinical manifestation (86.5%)^2^ makes it difficult to collect secretions to perform a local culture.

The observed results showed that clindamycin was the antibiotic of the first and second choice that was most often used for the treatment of secondary bacterial infections caused by snakebite. Therapeutic recommendations for antibiotic therapy include antimicrobials with activity against gram-negative, gram-positive, and anaerobic bacilli, such as chloramphenicol and amoxicillin clavulanate^8^. Patients with other bacterial soft tissue infections, such as erysipelas, should be treated with penicillin, and the dose and route of administration should be decided according to the severity of the case^8^. Data on bacterial diversity derived from abscesses and the effectiveness of the use of gentamicin or penicillin G for possible control have already been described^14^. In another study, high sensitivity of bacteria to amikacin, gentamicin, chloramphenicol, and sulfamethoxazole-trimethoprim has been identified^10^. However, in the protocols of the Infectious Diseases Society of America^8^, chloramphenicol is recommended for use in the treatment of secondary infections, which corroborates the results of other studies that demonstrate an extremely variable sensitivity of microorganisms to different antibiotics^9^ ^,^ ^10^ ^,^ ^14^ ^,^ ^15^.

The evolution of secondary bacterial infections in the skin can manifest itself through the presence of cellulitis and abscess, and drainage and debridement are recommended according to the needs of each case. These were, in fact, the most commonly used surgical techniques in the patients analyzed in this study. 

This study presented some limitations, since when performing an analysis using medical records, there is always the risk of lack of important information from clinical and laboratory evaluations. Another limitation was the lack of identification of the bacteria involved in the secondary infection, since, in most cases, the treatment was conducted empirically.

Snakebites that evolved into secondary infection were more common in the lower limbs, and most envenomations occurred in rural areas and were classified as moderate. The majority of patients received *Bothrops* antivenom therapy. Blisters were the elementary lesions presented by the majority of patients, with purulent exudate and necrotic tissue. The most commonly used antibiotic was clindamycin, which presented less therapeutic failures, and abscess drainage and debridement were the most commonly used surgical techniques. 

The results of this study demonstrate the importance of implementing specific guidelines aimed at standardizing the clinical management of these victims and considering randomized clinical trials that compare different antimicrobial treatments based on the profile for infections caused by snakebites.
